# Cultural, ethical, legal, and social considerations in genomics research with Indigenous Peoples: A scoping review

**DOI:** 10.1038/s41431-026-02065-2

**Published:** 2026-03-10

**Authors:** Rubi-Jayne Cohen, Rebekah McWhirter, Lyndsay Newett, Emily Colonna, Azure Hermes, Alex Brown

**Affiliations:** 1 National Centre for Indigenous Genomics, College of Science & Medicine, The Australian National University, Canberra, ACT Australia; 2 ANU College of Law, Governance and Policy, The Australian National University, Canberra, ACT Australia; 3 Yardhura Walani, National Centre for Epidemiology and Population Health, The Australian National University, Canberra, ACT Australia; 4 The Kids Research Institute, Adelaide, SA Australia

**Keywords:** Genetics research, Personalized medicine, Ethics

## Abstract

Indigenous communities are under-represented in genomics research, contributing to inequitable health-related knowledge, outcomes, and benefits. Under-representation reflects enduring consequences of colonial research practices that have engendered cultural, ethical, legal, and social (CELS) concerns among communities. Researchers must understand, navigate, and address these in their research practices. This study aimed to identify and synthesise CELS considerations to inform Indigenous genomics research practices. A systematic scoping review was conducted, including peer-reviewed papers on genomics that discussed cultural, ethical, legal, or social matters relevant to Indigenous Peoples globally; available in full-text and in English. Inductive content analysis using NVivo 12 Plus was undertaken to identify CELS considerations and develop content categories, with papers coded to multiple categories where relevant. As of May 2024, 186 papers were identified for inclusion: *n* = 70 (38%) included cultural, *n* = 91 (49%) ethical, *n* = 49 (26%) legal, and *n* = 125 (67%) social considerations. Cultural considerations included cultural harm, significance of blood, and the need to integrate Indigenous knowledges. Ethical considerations included consent, data access and sharing, privacy, and confidentiality. Legal considerations included laws protecting Indigenous interests, control of genomic samples and data, biovalue and DNA as a commodity, genetic discrimination, and the use of genomic data in constructing and defining racial identity. Social considerations included collective decision-making, genetic determinism, and stigmatisation, and the importance of contextualising findings within wider social determinants of health frameworks. Overall, researchers need to understand, navigate, and address CELS considerations of relevance to Indigenous Peoples to build trust, promote inclusion, and support equitable benefit-sharing in genomics research.

## Introduction

Indigenous Peoples are underrepresented in genomics research globally [1]. This under-representation reduces the potential for equitable benefit to arise from the knowledge and health-related outcomes possible with genomics research [2]. The reason for this under-representation, is in large part, due to mistrust, scepticism, and apprehension relating to genomics research, which stems from colonial research practices, and a lack of culturally-appropriate engagement and consultation with Indigenous Peoples [3].

Indigenous communities are diverse and often culturally and socially heterogeneous, with many groups maintaining traditional Knowledge Systems and practices; complex and interconnected social structures; diverse languages; and deep spiritual connection to ancestral homelands [4]. For the purposes of this manuscript, culture is defined broadly to encompass the knowledge, beliefs, values, and practices of Indigenous communities which shape their perspectives on, and participation in, genomics research.

Consistent for many Indigenous Peoples is a shared history of colonisation; displacement; dispossession of land, culture and language; exploitation, and systemic discrimination; and racism [5]. The ongoing impacts of colonisation are evident in enduring social, economic, and health disparities, as well as the colonial research practices that have contributed to and sustained these [6].

Efforts to address social, economic, and health disparities for Indigenous Peoples include international, national, and regional initiatives, strategies, programs, and policies to address social determinants of health, improve health outcomes, and ensure equitable access to healthcare. Foundational among these is the United Nations Declaration on the Rights of Indigenous Peoples (UNDRIP, 2007) which sets out individual and collective rights of Indigenous Peoples globally, including rights to maintain, control, protect, and develop their cultural heritage, traditional knowledge, and genetic resources, such as genetic data [2].

Health research, including genomics research, has an important role to play in addressing health disparities. Currently, there is limited knowledge regarding genomic variation within Indigenous populations, hampering the identification of biomarkers for early detection and the treatment of some diseases, and reducing the utility of, and access to, precision medicine [7]. Consequently, promoting inclusion in genomics research is essential, but insufficient in and of itself, to enhance equitable access to the health-related benefits of genomics research. For equity to occur, researchers must build trust with Indigenous communities. However, before engaging with Indigenous communities, researchers need to understand the CELS landscape that underlies genomics research and use these understandings to inform and improve research practice with, and across heterogeneous Indigenous communities.

A scoping review of the global literature was conducted to gain an understanding of CELS factors pertinent to genomics research involving Indigenous Peoples, and to develop key considerations to assist both Indigenous and non-Indigenous researchers to engage with, account for, and incorporate these considerations into any genomics work. Findings are intended to inform and guide research practice, support trust building, and promote inclusion and equitable benefit.

## Methods

The authors recognise that individual and collective worldviews and experiences influence perspectives, and that author standpoints impacted how the review was conducted, and findings interpreted. Our team brings Aboriginal lived experience (R.C, A.H, A.B.) and experience in genomics, sociology, public health, epidemiology, and decolonising, strengths-based methodologies (all authors).

### Eligibility criteria

Papers were included if they were peer-reviewed, discussed CELS matters related to genomics research involving Indigenous Peoples, available in full-text, and in English. To narrow focus to contemporary health-related genomics research, papers on ancestry testing, ancient DNA, and migration were excluded.

### Information sources

Databases searched were Scopus, Web of Science, ProQuest, and Google Scholar. All papers available on the databases on 9 May 2024 were included.

### Search strategy

The following search string was entered into each database:

((Aborig*) OR (“Torres Strait Islander*”) OR (Indigen*) OR (Māori) OR (“Native American”) OR (Metis) OR (Inuit) OR (“First People*“) OR (“First Nation*”)) AND ((Genom*) OR (DNA) OR (genet*) OR (gene) OR (genes)) AND ((Social) OR (Society) OR (Ethic*) OR (Legal*) OR (ELSI) OR (CELSI) OR (Cultur*)).

The review followed the PRISMA Extension for Scoping Reviews (PRISMA-ScR) checklist [8].

### Screening of evidence and data charting

**Fig. 1:**
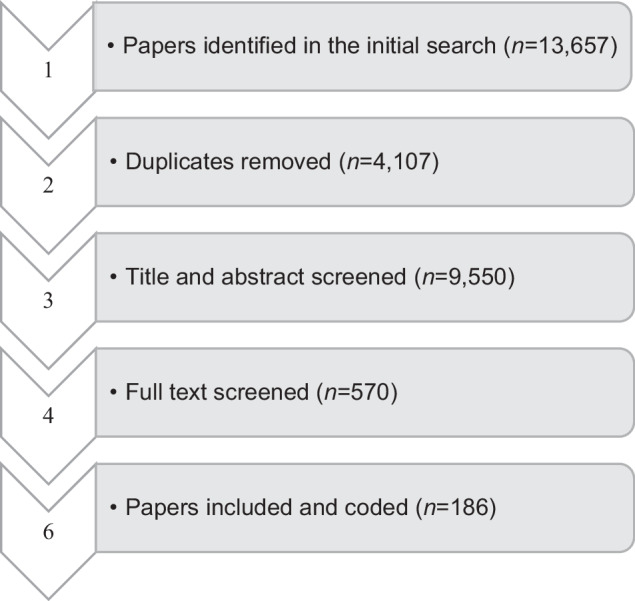
PRISMA flow diagram [8]. Flow diagram outlining the study identification and screening process. The number of papers at each stage is indicated in parenthesis (*n*). A total of 13,657 papers were identified through the initial search, 4107 duplicates were removed. A total of 9550 titles and abstracts were screened, 570 full-texts were screened for eligibility, and 186 papers were included and coded in the final analysis.

Sources were collated in Endnote and imported into Covidence. The search string yielded an initial 13,657 papers. Covidence and manual screening excluded 4107 duplicates. Title and abstract screening excluded 8890 papers. Full-text review excluded 384 papers. See Fig. 1.

The following information for each paper was recorded in Excel (Appendix 1, Table 1):

1. author(s),
2. year of publication,
3. study location/context for paper, and
4. indication of whether paper included discussion of a CELS matter.

### Analysis

A total of 186 PDFs were imported into NVivo 12 Plus for analysis. Papers were analysed using an inductive content analysis approach that aligned with steps outlined by Vears and Gillam (2022). This approach was chosen for its suitability for analysing health-related data [9] and involved five iterative steps:

1. Reading and familiarisation with the data.
2. First-round coding to capture all references to cultural, ethical, legal, or social considerations.
3. Second-round coding to refine and consolidate the initial codes.
4. Refining sub-categories within each CELS domain.
5. Synthesising and interpreting the key themes.

References to cultural practices, traditions, values, and beliefs were coded, and grouped to form ‘cultural considerations’. For ‘ethical considerations’, codes, words or phrases related to consent, privacy or confidentiality, and data access and sharing. For ‘legal considerations’, codes included references to laws, legislations, frameworks, ownership, and control of samples and or data. Finally, for ‘social considerations’ codes included references to, collective decision-making, stigmatisation, and genetic determinism and social determinants of health. Since many papers considered multiple aspects of cultural, ethical, legal, and social factors, each paper could be coded to more than one CELS category.

## Synthesis of results

### Selection of sources of evidence

Papers were reviewed for inclusion at each stage by two authors (R.C., E.C.) independently, with disagreements resolved through regular in-person consultation. This resulted in a final set of 186 papers published between January 1997 and May 2024. Included papers discussed one or more CELS issues within the context of genomics research involving Indigenous Peoples. A diagrammatic representation of the screening process is shown in Fig. 1.

The search criteria captured Indigenous populations of Africa (*n* = 2), Australia (*n* = 28), Brazil (*n* = 3), Canada (*n* = 12), the Caribbean (*n* = 1), Chile (*n* = 2), Italy (*n* = 1), Laos (*n* = 1), Latin America (*n* = 1), Mexico (*n* = 2), New Zealand (NZ) (*n* = 19), Norway (*n* = 1), Peru (*n* = 3), Philippines (*n* = 1), South Africa (*n* = 5), South America (*n* = 1), Taiwan (*n* = 1), United States (US) (*n* = 58), Venezuela (*n* = 1), and West Africa (*n* = 1). Some papers captured Indigenous populations from multiple (2–31) countries (*n* = 42) (see also Table 1 in supplementary materials).

As the review was designed to capture a global perspective, the terms ‘Indigenous communities’ and ‘Indigenous Peoples’ are used respectfully, recognising that this includes diverse and heterogeneous peoples. Where specific peoples are referred to, more specific terminology is employed.

### Cultural considerations

**Fig. 2:**
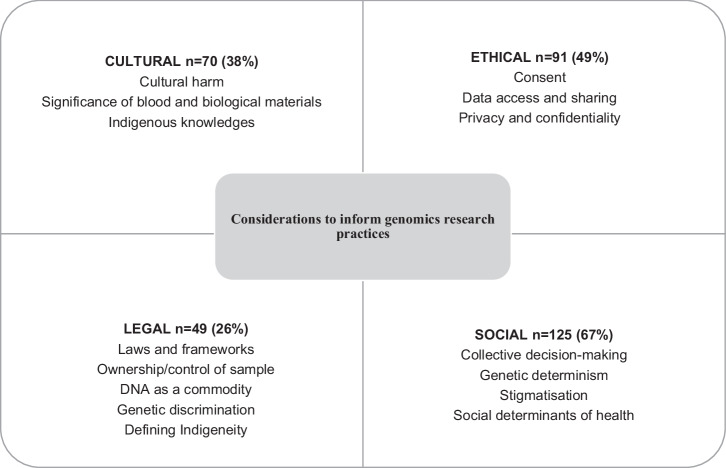
Overview of broad CELS content categories developed. Diagram summarising the four major CELS domains and their frequency of occurrence in the reviewed literature. The number (*n*) of papers addressing each domain is shown, with percentages representing the proportion of included studies (*N* = 186). Cultural (*n* = 70, 38%) includes cultural harm, significance of biological materials, and Indigenous knowledges. Ethical (*n* = 91, 49%) includes consent, data access and sharing, and privacy and confidentiality. Legal (*n* = 49, 26%) includes laws and frameworks, ownership/control of samples, DNA as a commodity, genetic discrimination, and defining Indigeneity. Social (*n* = 125, 67%) includes collective decision making, genetic determinism, stigmatisation, and social determinants of health. The central box indicates the overarching aim of informing genomics research practices.

As Fig. 2 shows, over a third (38%) of papers discussed cultural considerations relevant to informing genomics research practices. Three cultural content categories: cultural harm resulting from genomics research; the significance of blood and biological materials; and integrating Indigenous knowledge with Western science, were identified.

### Cultural harm and challenges to Indigenous histories

Genomics research can cause cultural harm by producing narratives that conflict with rich and deeply held oral traditions that function to transmit history, culture, and identity across generations [10]. For example, many Indigenous Australians believe their ancestors have always been on the land, and creation stories are deeply tied to the land itself. These cultural and spiritual beliefs are passed down through generations and often linked to the concept of the Dreamtime which describes the time when ancestral beings created the land, animals, and people [11]. This can sit uncomfortably with genomics research which indicates that all modern humans originated in Africa and migrated to other parts of the world - including Australia - and implies Indigenous Australians only arrived in Australia tens of thousands of years ago. The divide between scientific evidence and Indigenous histories can undermine cultural and spiritual connections to land, affect claims to land and resources, disrupt cultural identity, cause distress and alienation, and denies sovereignty [12, 13]. To account for this consideration, researchers should work respectfully with communities to understand beliefs and cultural values so as to anticipate and mitigate potential conflicts, particularly in the framing and dissemination of research findings [11].

### Significance of blood and biological materials

The need for culturally informed and sensitive research is especially important in research involving the collection of blood and other bodily tissues, which are often viewed as symbolic, spiritual, sacred, and intertwined with identity [2, 14–16]. For example, for some Native American tribes, blood is intertwined with identity and group membership, making its collection for research purposes highly sensitive [16]. This is evident in the unwillingness of some Navajo people to accept blood transfusions from non-Navajo individuals due to concerns about maintaining cultural and political boundaries [16]. Misunderstandings or disregard for cultural beliefs can cause Indigenous communities to view researchers with distrust, and associate research methods with exploitation which can exacerbate cultural tensions [14]. These cultural beliefs require that researchers take care when selecting and employing research methodologies, so values are respected, and harms are mitigated [17].

### Integrating Indigenous worldviews into research

Researchers and Indigenous communities may differ in their sociocultural contexts, making it challenging to anticipate and address research-related risks [18]. Additionally, Indigenous communities may prioritise risks that seem inconsequential to researchers, particularly when these relate to cultural beliefs and social structures [19]. For example, collecting family histories might be considered straightforward for some researchers but can involve complex structures, and community-specific rules or taboos, such as the mentioning of deceased family members’ names among some Indigenous communities [20]. Furthermore, the concepts of ‘kin’, ‘relatives’ (or relations) and ‘family’ are bound within cultural conceptions which can extend beyond biological relationships [21].

The successful integration of Indigenous knowledge and Western science requires approaches that respect cultural beliefs and values. This notion is captured by the concept of ‘two-eyed seeing’, a philosophy that promotes seeing the strengths of Indigenous Knowledge Systems and Western scientific approaches; summarised as “…learning to see from one eye with the strengths of Indigenous knowledges and ways of knowing, and from the other eye with the strengths of Western knowledges and ways of knowing, and to using both these eyes together, for the benefit of all.” [22].

This﻿ kind of integration can be achieved by including Elders and knowledge holders early in research planning to advise on cultural protocols [20]; through the establishment of community partnerships [22]; and with the prioritising of community-determined principles. For example, principles among Māori that encompass ownership, consent, and the spiritual integrity of biological materials - like *tikanga* - have been shown as essential for ensuring cultural safety in Māori research [23].

**Key considerations for culturally informed and collaborative research practices**

- Work with local researchers to gain community-specific knowledge before data collection, and incorporate traditional knowledge, when appropriate, into research design.
- Establish an understanding of cultural beliefs that allow research findings to be framed and disseminated in ways that respect cultural narratives.
- Carefully consider and adapt research methodologies to respect Indigenous Peoples and communities values and to protect against group harm.

### Ethical considerations

Ethical considerations were highlighted in almost half (49%) of all reviewed papers. Three content categories were demarcated: consent; data access and sharing; and privacy and confidentiality. Within the latter two categories, considerations relating to secondary data use, and population size, were deemed prominent.

### Consent

Informed consent is a foundational principle in research ethics that ensures participants fully understand the nature of their involvement, and can voluntarily participate without coercion [24]. Researchers must obtain informed consent from participants before conducting research [25, 26], with UNDRIP stating that *free*, *prior*, informed consent (FPIC) must be explicitly granted through consultation with Indigenous participants [27]. Despite its significance, obtaining informed consent remains difficult. Misunderstandings of genomics concepts by participants, a lack of cultural sensitivity among researchers, historical mistrust, and misalignment between consent models’ and Indigenous values and governance structures are not adequately accounted for [15, 28–30]. Identified challenges in obtaining informed consent also include: language barriers, educational barriers, and inadequate explanations of research concepts [25, 30]. For example, genomics research terms may not have direct equivalents in Indigenous languages, causing misunderstandings, and lack of clarity, which compromise the legitimacy of consent [25, 30]. Communities where local Indigenous languages are primarily spoken, and/or where there is less experience with scientific research projects and genomic science concepts - whether due to limited exposure or inadequate awareness raising - may not fully comprehend implications of involvement in genomics research, and therefore, be unable to provide FPIC. This can leave communities potentially vulnerable to exploitation, even if inadvertently [14]. Researchers must anticipate and address consent-related challenges to be able to appropriately communicate the purpose and perceived risks of their research to ensure FPIC is obtained.

#### Community and collective consent

Several papers emphasised the importance of community or collective consent, alongside individual consent. Collective consent acknowledges that genomics research has implications beyond the individual and as such should involve approval from community, tribal or kin groups to demonstrate respect for local governance and decision making structures [23, 31]. Examples of well conducted collective consent include Māori research frameworks that integrate family, and community engagement, and recognise that consent is shaped by interrelationships within kin groups, and the broader community. As Beaton et al (2015) highlight, “the interrelationship between individuals and family or kin groups … forms the cornerstone of establishing a “collective” consent” [23]. Additionally, memorandums of understanding (MOUs) are formal agreements developed through community consultation that document researcher responsibilities and expectations, and operate as an endorsed form of collective consent or community approval [32]. Researchers should obtain collective consent from local governing community organisations before seeking individual consent, and this collective consent should be secured before approaching national or jurisdictional ethics committees [33].

#### Abiding by original consents and the importance of reconsent for secondary use of data

Rapid changes in scientific research can pose challenges for adhering to original consent agreements, with some research guidelines acknowledging that future uses of samples cannot always be anticipated at time of collection [18]. Meanwhile, the secondary use of samples and data without reconsent (e.g. by relying on broad consent or utilising mechanisms for waivers of consent) denies individuals the ability to withdraw, and/or to manage how their samples and data are used, and for what purposes [34]. This conflicts with the principle of self-determination which, in the genomics research context, supports the authority of Indigenous Peoples to decide how their genomic data is collected, stored, and used [34]. While the principle of self-determination is recognised internationally through UNDRIP, the ability of Indigenous communities to govern their research data varies widely, and in some contexts, formal mechanisms for data governance may be limited or absent [35].

A comparative study of Native Hawaiian and non-Hawaiian preferences for informed consent found Native Hawaiian respondents were significantly more likely to require informed consent for secondary use of samples [17]. These preferences were influenced by religion, sex, traditional beliefs, historical discrimination, and social harm that have contributed to distrust in genomics research [17]. These findings suggest that consent for secondary use of samples may be even more important for Indigenous research participants due to past colonial research practices and a legacy of mistrust. Challenges associated with original consent and reconsents underscore the importance of maintaining ongoing relationships and communication with participants and communities to ensure continued consent for future use of samples and data [18]. Anticipating and navigating these challenges, and ensuring new studies align with the original scope of consent, actively seek reconsent, and/or employ dynamic consent approaches can redress unethical research practices, and support trust and inclusion in genomics research [10].

### Data access, sharing and indigenous data governance

Data access and sharing via open data models like ‘broad access’, ‘open access’ and ‘available upon request’ access are promoted by major funders, including the European Research Council (ERC), National Institutes of Health (NIH) in the US, and the National Health and Medical Research Council in Australia (NHMRC), to support free availability and maximise use of publicly-funded research data for public benefit [36]. In the US, NIH policy even places expectations on researchers to obtain broad consent rather than specific consent, which is often in conflict with community preferences [37]. These models allow access to genomic data without explicit community, or individual consent, for secondary purposes. This undermines principles of community-based participatory research, and has been criticised for leaving communities vulnerable to exploitation and misuse [31, 32]. Furthermore, these models undermine Indigenous Data Sovereignty which emphasises the right of Indigenous communities to govern and control how data is used and shared [1, 32]. In opposition, communities are advocating for culturally sensitive, restricted, or controlled access to protect both cultural integrity and intellectual property [32].

Ethical considerations around data access and sharing in genomics research with Indigenous communities revolve around balancing scientific advancement with Data Sovereignty, and the rights Indigenous Peoples have to govern research, and control access to data about them. While the ERC promotes the FAIR data principles of ‘findable’, ‘accessible’, ‘interoperable’ and ‘re-usable’, it notes that not all data can, or should, be made fully open for privacy or security reasons, and encourages researchers to justify data restrictions in their management plans [36]. Similarly, the Global Alliance for Genomics and Health (GA4GH) promotes responsible data sharing through frameworks that align with the FAIR principles, while supporting data governance models that respect Indigenous rights, including community-led decision-making and culturally appropriate consent processes [38]. Ensuring community control, addressing historical exploitation, and respecting Data Sovereignty are crucial to culturally appropriate, and ethical research. Governance models like federated learning and frameworks such as Ownership, Control, Access, and Possession (OCAP) in Canada and Collective Benefit, Authority to Control, Responsibility, and Ethics (CARE) principles offer promising strategies for embedding Indigenous Data Governance into genomics research [1, 37]. Ensuring that genomics research aligns with Indigenous Data Governance principles requires the ongoing adaptation of policies and research practices to prioritise community control and consent.

### Privacy and confidentiality

For many Indigenous communities, privacy and confidentiality are complicated by socio-cultural norms that must be accounted for in research frameworks. For example, among Māori, collective well-being is prioritised over individual autonomy. In fact, traditionally, health issues were addressed collectively through open sharing of knowledge to problem solve; a practice that continues to inform approaches to research privacy and confidentiality [23]. The Māori concept of *whakapapa* (genealogy), therefore, can be seen to conflict with a Western notions of research confidentiality [39]. These considerations are not unique to Māori, with many Aboriginal communities in Australia and First Nations communities in Canada holding collectivist views on privacy, with health information deemed a collective responsibility [40]. By familiarising themselves with local research frameworks, researchers can design methodologies that reflect community understanding of privacy and confidentiality; including who has access to information and how it is shared.

#### Population sizes

Related to confidentiality is the susceptibility of some communities to breaches of privacy due to small populations sizes [34]. Some papers highlighted the enduring consequences of colonisation and dispossession resulting in small population and community sizes. Consequently, even when researchers attempt to anonymise or deidentify variables, community members in small populations may still be able to identify each other, posing risks to privacy that must be appropriately mitigated in research protocols [34, 41]. These risks may remain even when research complies with all relevant legal obligations regarding privacy.

**Key considerations for ethical research to facilitate Indigenous Data Sovereignty and Governance**

- Ensure the consent model utilised aligns with Indigenous values, existing governance structures and UNDRIP, and work to maintain ongoing relationships with communities.
- Support community control, respect Data Sovereignty and utilise appropriate governance models.
- Incorporate local frameworks and ways of doing into research methodologies to ensure proper privacy and confidentiality.
- Detail how small population sizes will be accounted for to protect privacy in research protocols and agreements.

### Legal considerations

Legal considerations were discussed in a quarter (26%) of papers. Five content categories were developed: laws protecting Indigenous Peoples interests; ownership and control of genomic samples and data; biovalue and DNA as a commodity; genetic discrimination; and constructing and defining racial identity.

### Laws protecting Indigenous interests

The ongoing development of technologies and scientific research involving human biological material has necessitated the enactment of legal instruments to protect Indigenous Peoples [34]. Several international and national legal frameworks address these issues. A selection of frameworks relevant to genomics research involving Indigenous Peoples is summarised in Appendix 1, Table 2 [3, 5, 6, 15, 23, 37, 42–44]. When undertaking genomics research, it is important that researchers are familiar with relevant legal frameworks.

### Ownership of sample and data: collective ownership, donation vs. DNA on loan and individual protections

Genomics research involving Indigenous Peoples raises significant legal implications regarding genomic data ownership and control. Here we refer to considerations of property law, contract, and other legal mechanisms that give effect to the ownership or control of genomic samples and data, such as formal agreements governing sample collection, storage, and use. Many Indigenous groups assert that genomic information is the collective property of the community it was obtained from, and emphasise that the community should have collective oversight of samples and data, which is reflective of Indigenous Data Sovereignty principles [34]. The conceptualisation of genomic information as personal property is largely a Western notion, which can conflict with collectivist worldviews and cultural protocols. This discrepancy has necessitated the establishment of tribal and community review boards, committees, and research agreements for data sharing, to ensure data is returned and appropriately governed [5, 34, 37].

Discussions of ownership related to sample collection were described in different ways using the terms: participant, donor, sample contributor, altruistic, commodity, donation, gift, and ‘DNA on loan’. Two main models were dominant in the literature: ‘DNA as a gift or donation’ and ‘DNA on loan’ [18]. The former model considers samples to be gifts, given by individuals or communities, and is a model that has been used in research projects with Māori to align with cultural values. For example, the Aotearoa Variome project contextualised that DNA taken was *takoha*, a gift of responsibilities, that bestowed and obligated whoever used the data to deliver useful outcomes for Māori [45]. DNA as a gift or donation is a model common in the US but it does not have the same association with *takoha* or gift of responsibilities. Instead, it implies a transfer of property rights from donor to researcher or institution [3].

In contrast, DNA on loan - promoted by the Canadian Institutes of Health Research Guidelines for Health Research Involving Aboriginal People - is a custodianship model, with samples remaining the property of that person or community, and being only temporarily entrusted to researchers, who function as custodians [42, 46]. By treating samples as temporarily entrusted to researchers, the DNA on loan model protects communities against re-use without permission, reinforces community ownership, and helps to foster trust in genomics research [31, 37, 42, 46]. DNA on loan upholds principles of respect and reciprocity which are integral to many guidelines and frameworks developed to ensure ethical and culturally appropriate research [42]. To account for different understandings of ownership, researchers must consider, and communicate participant rights in their approaches, and negotiate locally appropriate mechanisms and methods to align with cultural protocols.

### Biovalue and DNA as a commodity

In the context of genomics research, ‘biovalue’ refers to the value derived from biological materials, particularly DNA, and its associated data [31]. Advances in technologies and privatised research has allowed the genomic data of individuals and distinct collectives, such as Indigenous groups, to become commodities through bio-patents and pharmaceuticals [43, 47]. Patenting human genetic materials is allowable under current law in some countries. For example, in the US, the Supreme Court case, Diamond v. Chakrabarty, 477 U.S 303 (1980) ruled that genetically engineered bacterium could be patented as they constituted a new and useful invention [48]. The European Union Convention allows the patenting of genetic material if the criteria of novelty, inventive step, and industrial applicability are met. Under the Biotech Directive (98/44/EC) human genes and gene sequences are patentable if they are isolated from their natural environment and provide specific, substantial, and credible utility [49]. These legal precedents do not align with Indigenous worldviews, with some Indigenous groups asserting genetic data derived from biological samples “are part of a life form which cannot be claimed as invented” [50]. Yet, there have been multiple instances of applications for genomic patents related to biological materials derived from Indigenous People without appropriate, prior consultation and consent, and with blatant secondary use of data without permission [51]. Some Indigenous People believe DNA and resultant data should be treated as economic commodities with compensation provided, especially if research yields profits [31]. This view is influenced by historical exploitation, enduring social inequities and unethical past research practices. As the following excerpt from a 2002 consent form shows, it is also a warranted position [52]:

*Discoveries made with your DNA samples may be patented by us and the University. These patents may be sold or licensed, which could give a company the sole right to make and sell products or offer testing based on the discovery. Royalties may be paid to us, the University, and the Sponsor. It is not our intent to share any of these possible royalties with you* [43].

In contrast, Foster, Bernsten and Carter (1998), illustrate how researchers can share economic benefits with the people who facilitate them, by detailing work with an Apache community review board to draft a research agreement that enabled the allocation of a portion of royalties to the tribe to support health and education [53].

*The university will deduct 10% of royalties for legal and administrative costs. Of the remainder, the university will retain 30%, the tribe will receive 30%, and investigators will receive 30%. The unassigned 10% will be retained in a reserve fund for liability or litigation. The Apache CRB [community review board] resolved that any royalties to the tribe be earmarked for the promotion of the health and education of tribal members* [53].

Agreements such as these are uncommon because existing legislation and research governance frameworks often do not include formal mechanisms for community oversight. To ensure research benefits Indigenous communities, agreements of this kind must be prioritised.

### Genetic discrimination

Genetic discrimination occurs when people are treated unfairly because of presumed genetic traits, often associated with insurance, employment, and bias in forensic databases. Genomics research has the potential to contribute to genetic discrimination, and to exacerbate existing inequalities, especially in relation to accessing health insurance, life insurance, and employment [12, 31, 54]. Indigenous Peoples share concerns that genetic predispositions to health conditions could be used against them, and that findings could reinforce harmful racial stereotypes and further stigmatise, discriminate, and exacerbate inequalities in accessing health and life insurance [31, 55, 56]. The risk of genetic discrimination resulting from genomics is recognised internationally in the proposed legislation banning the use of genetic results in life insurance underwriting in Australia, and enactment of the Genetic Information Non-discrimination Act (GINA) in the US [14]. Upholding the principle of FPIC is therefore critical as it ensures participants understand potential risks, including discrimination, and can make informed decisions about their participation in genomics research. This requires researchers to remain aware of jurisdiction-specific risks associated with participation in genomics research, and to communicate these transparently to participants.

### Constructing and defining racial identity

Genomics research has been used to construct racial identities and group membership which can have legal implications, and is in tension with the notion that race is a social construct, with group membership defined by socio-cultural parameters [6, 57]. The US maintains a form of biologically determined group identification, ‘blood quantum’, which is a measure of Native American ancestry that can be used to determine group membership and allocation of resources by governments and tribes [13]. Introduced as a colonial measure, blood quantum is still used by some Native American tribes, with some members concerned that, used in this way, genomics can be a “legal Pandora’s box” that can benefit some and exclude others [13]. Laws or research that looks to construct racial identities using genomics undermines the three-part determination of Indigeneity – descent, self-identification, and community recognition – used throughout much of mainland Australia [57]. Beyond the US and Australia, other regions define race and Indigenous identity based on social, cultural, and historical factors, highlighting the necessity for researchers to understand, navigate, and tailor research methodologies to the Indigenous community they intend to engage with.

**Key considerations for legal responsibilities in Indigenous genomics research**

- Be familiar with international, national and local laws, legislations, and frameworks that prioritise Indigenous Peoples.
- Prioritise transparency when it comes to participants’ legal rights to samples and resultant data, particularly in relation to research protocols and agreements.
- Understand and communicate the risks of being involved in genomics research and what this may mean for insurance and employment.
- Engage with communities early to discuss potential benefits arising from research, including intellectual property, data use, and benefit-sharing agreements.
- Understand how identity is understood and framed within these contexts.

### Social considerations

Two-thirds (67%) of the papers included discussion of social considerations, with analysis leading to three content categories: the importance of collective decision-making; the potential for genetic determinism and stigmatisation; and the need to focus on social determinants of health to contextualise findings.

### Collective decision-making processes

Indigenous cultures and communities often practice collective decision-making, reflecting collectivist worldviews [58]. These practices contrast with individualistic Western approaches where research practices align with an individual’s right to decide, rather than a group’s collective decision-making process [6]. For many Indigenous groups, genomic material is viewed as belonging to the collective, and not something that an individual can consent to donate or sell [29]. Consequently, providing samples for research may require community agreement alongside individual FPIC [29]. In Australia, many Aboriginal communities adhere to communal or hierarchical decision-making, reflecting deep respect for decision-making community leaders, including Traditional Owners and community Elders [6]. Researchers should demonstrate respect for collectivist views and abide by existing community decision-making processes.

### Genetic determinism and stigmatisation

Genomics research is also linked to genetic determinism and stigmatisation. Genetic determinism suggests a person’s genes determines their characteristics and behaviours, including health and intelligence [4, 31, 55, 59, 60], and can adversely impact Indigenous communities; particularly by oversimplifying complex influences on disease, health, and behaviour [31, 55, 59]. An example is the controversial Warrior Gene hypothesis which posited that presence of the monoamine oxidase A (MAOA) gene in male Māori participants explained high rates of aggression, crime, and incarceration [60]. Another example is the thrifty-gene hypothesis applied to Indigenous Peoples in Canada and Australia to argue for a biological pre-disposition for Type-2-diabetes caused by increased fat storage during times of famine that had evolutionary advantage in the past [61]. Despite their popularity, no empirical data has yet confirmed these hypotheses. In both cases, researchers failed to account for the history of colonial oppression and enduring inequality experienced by Indigenous populations. Such narratives and framing of complex social issues as genetically inevitable shift blame from structural and historical factors and reinforce stigma. Associating genetic traits with Indigenous populations can result in broad stigmatisation that affects entire communities, disrupts social structures, and causes tensions and psychological distress [30].

At the same time, genomics research has the potential to de-stigmatise health conditions and improve healthcare. For example, in NZ, research with the Ngāti Porou people identified genetic variants in gout which contributed to de-stigmatising the condition by showing that it is hereditary, and not due to lifestyle choices [45]. This understanding improved treatment, and allowed Māori to consume traditional foods without fear of gout attacks [45]. To address this risk of genomics research, researchers must recognise and outline the potential for genetic determinism and stigmatisation, as well as taking care to present findings in ways that are strengths-based. This may be achieved through collaboration with involved communities in the process of drafting and disseminating research findings.

### Social determinants of health

The focus of genomics research also means other factors that contribute to health such as the social determinants of health; specifically, socioeconomic status, education, healthcare access and environment can be neglected, or ignored; despite patterning exposures to health risks shaping health outcomes, and driving disparities [55]. The relevance and impact of the social determinants of health is evident in the work of Riegos and Azcorra-Pérez (2023), which describes how the historical experiences of the Maya people of Mesoamerica caused structural inequalities, distanced them from protective traditional diets, reduced social cohesion, and increased stress, which are all risk factors for metabolic diseases like obesity, and diabetes mellitus. The authors demonstrate a deep understanding of the interplay between historical experiences, genetic considerations and social-cultural and environmental contexts, and how these shaped contemporary health among Maya [15, 55, 62–64]. Considering social determinants of health when analysing genomics research findings can avoid the oversimplification and misrepresentation of health issues, and perpetuation of stigmatising and genetic determinist research [7, 15, 29, 55, 62].

**Key considerations for social and community contexts in genomics research**

- Respect and account for established community decision-making processes.
- Communicate the nuanced relationship between genetics, environment and disease and any foreseen risks of stigmatisation and purposefully adopt strengths-based framing for findings where possible.
- Conduct contextually relevant analysis of social determinants, with appropriate emphasis to prevent deflection from existing social health disparities.

## Discussion

This systematic scoping review aimed to identify considerations researchers need to recognise, understand, and address to ensure research with Indigenous Peoples is culturally, ethically, legally, and socially informed. Inductive content analysis of 186 papers facilitated the identification of considerations to inform practice (summarised in Table 1). Table 1 distinguishes between what researchers should understand about each domain before, and during engagement with Indigenous communities, and the actions they can take to conduct research that is culturally safe, ethical, legally compliant, and socially responsible.

**Table 1:** Key considerations for CELS informed research practice.

Key considerations to inform CELS research practice		
What should researchers understand?		What should researchers do?
**Culturally informed research**	• Potential conflicts between Indigenous narratives/traditions and genomics research. • Local cultural contexts to avoid group harm and misinterpretation. • Interplay of physical, cultural, spiritual, family, and community spheres.	• Frame research findings to respect cultural narratives. • Adapt methodologies to respect Indigenous values and protect against group harm. • Engage local researchers to gain community specific knowledge before data collection and incorporate traditional knowledge, when appropriate.
**Ethically informed research**	• Historical, social, and cultural contexts of Indigenous communities. • Balance between scientific advancement and Indigenous rights regarding data access and sharing. • Existing governance models for data protection. • Necessity of voluntary, explicit consent for primary and secondary use of samples and data. • Privacy concerns in small populations.	• Ensure consent models align with Indigenous values, governance structures, and UNDRIP. • Maintain ongoing relationships with communities. • Support community control, respect Data Sovereignty, and utilise appropriate governance models. • Incorporate local frameworks into research methodologies to ensure privacy and confidentiality. • Account for privacy concerns in small populations.
**Legally informed research**	• Laws, legislations, frameworks, policies, and guidelines protecting Indigenous communities. • Collectivist perspectives around sample and data ownership. • Importance of co-developing research agreements for data control. • Value of collaborative community input to protect community interests and prevent harm. • Risks of participation, including for insurance and employment. • Local context and how Indigenous identity is understood.	• Familiarity with international, national, local, and Indigenous laws, legislations and frameworks. • Be transparent about participants’ legal rights to sample and resultant data. • Engage with communities early to discuss potential benefits, intellectual property, data use, and benefit-sharing agreements. • Communicate risks of participation, including for insurance and employment. • Incorporate local definitions of identity and collaborate with communities to respect these in study design and consent processes.
**Socially informed research**	• Importance of respecting community decision-making structures. • Nuanced relationship between genetics, environment, and disease to avoid genomic determinism and stigmatisation. • Contextually appropriate analysis to prevent reinforcing social health disparities.	• Respect community decision-making processes. • Communicate these nuanced relationships clearly and highlight potential risks of stigmatisation. • Use strengths-based framing for findings and carefully analyse social determinants to prevent deflection from existing disparities.

Culturally appropriate genomics research requires a deep understanding of local contexts and anticipation of potential group harms [19]. Collection of blood and bodily tissues must consider and account for cultural contexts to avoid misinterpretation and harm. This can be facilitated by incorporating Indigenous world views into research methodologies [14, 22].

In the context of genomics research involving Indigenous Peoples, consent is a complex issue that involves recognising the historical, social, and cultural context of communities, and ensuring balance between individual autonomy and collective decision-making [18]. To meet ethical expectations, consent must be voluntary and explicit for both primary and secondary use of samples and data, with clear processes established to guide decisions about unforeseen secondary use. Data access and sharing should balance scientific advancement with the protection of Indigenous rights. Federated learning and frameworks such as OCAP and the CARE principles offer promising strategies.

To navigate the genomics research space, researchers must understand the laws, legislations, frameworks, policies, and guidelines designed to protect the rights and interests of the Indigenous communities they are working with. Researchers must understand collectivist perspectives of ownership and co-develop research agreements to ensure communities have control over how data is used, stored, and shared now, and into the future. Inclusive and collaborative community input can protect against discrimination, protect community interests, and rights, and ensure research practices are respectful, culturally sensitive and have equitable benefit [12, 19, 24, 30, 41, 62].

Genomics research can negatively affect communities through cultural undermining, and dignitary harms; the perpetuation of social inequalities; and the reinforcement of stereotypes through genetic determinism, and stigmatisation [18, 20, 34]. Researchers must understand, respect and adhere to community-specific decision-making structures [33, 58]. By understanding and being able to transparently communicate the nuanced relationship between genetics, environment, and disease, researchers can avoid reinforcing genomic determinism and perpetuating stigma [55, 59]. Contextually appropriate analysis of social determinants, and even emphasising these determinants, can prevent deflection from existing social and health disparities [55, 62].

### Limitations

The search string included Indigenous groups known to the lead author (e.g. Metis) which may have skewed the articles identified in the databases and the analysis above. However, inclusion of broad terms like ‘First Nation’, ‘First people’ and ‘Indig*’ hoped to capture a global view of genomic research involving Indigenous Peoples. This is supported by capturing genomics research involving Indigenous Peoples from at least 31 countries. Additional limitations include the reliance on English language publications which may have excluded relevant papers from non-English speaking regions. Moreover, the diversity of Indigenous communities globally means the main findings may be more relevant to some contexts than others. The authors encourage interpreting and applying the findings with respect to each community’s specific context.

While this review aims to provide a high-level summary of relevant considerations, it is important to recognise that there exists substantial diversity between Indigenous Peoples, and that not all considerations identified here will be applicable in all locations or across all genomic contexts. Further, future research could usefully extend these findings by evaluating how specific considerations may generate benefits for Indigenous communities.

## Conclusion

Indigenous communities are under-represented in genomics research, resulting in inequitable health knowledge, outcomes and benefits. This under-representation is a consequence of historical and enduring colonial research practices, which foster mistrust and scepticism towards genomic science [4, 30, 62, 65]. These practices are unethical and extractive in nature. They disregard local contexts, knowledges, autonomy, practices and values, lack benefit; and are generally characterised by uneven power dynamics between researchers and Indigenous participants [14, 15, 29, 32]. Because of practices like these, Indigenous communities have unequal access to the health-related benefits of genomics research.

Researchers are charged with building trust to promote inclusion, and to support equitable benefit-sharing with respect to the knowledge and health-related outcomes that are possible with genomics research. Researchers must avoid perpetuating colonialist research practices, and therefore, must be guided by the cultural and social dynamics inherent to many Indigenous communities. To ensure appropriate international, national and local knowledges are guiding research practices, methodologies must be ethically and legally rigorous, and researchers must recognise their privilege to work in the Indigenous genomic space, and within diverse, multi-skilled teams.

## Supplementary information

Appendix 1, Table 1 & Appendix 2, Table 2

## Data Availability

The papers analysed are available from the corresponding author on reasonable request.
