# Supplementary material for: Cultural, ethical, legal, and social considerations in genomics research with Indigenous Peoples: A scoping review
Source: Eur J Hum Genet. 2026 Mar 10;34(6):749–58. doi: 10.1038/s41431-026-02065-2 (PMC13247151; doi:10.1038/s41431-026-02065-2)
Supplement: Supplementary file 1 — Appendix 1, Table 1 & Appendix 2, Table 2 [file 41431_2026_2065_MOESM1_ESM.docx]

Appendix 1

Table 1: alphabetised list of included papers and identification of CELS issue or consideration.

| # | **Authors** | **Year** | **Study location/context** | **Cultural** | **Ethical** | **Legal** | **Social** |
| --- | --- | --- | --- | --- | --- | --- | --- |
| 1 | Abadie et al. | 2015 | United States |  |  |  |  |
| 2 | Aguilar-Ordoñez et al. | 2022 | United States |  |  |  |  |
| 3 | Ahuriri-Driscoll et al. | 2021 | New Zealand |  |  |  |  |
| 4 | Allen | 2009 | Various or Global |  |  |  |  |
| 5 | Alper & Jon Beckwith | 1999 | Various or Global |  |  |  |  |
| 6 | Aramoana & Koea | 2020 | Various or Global |  |  |  |  |
| 7 | Arango-Isaza et al. | 2023 | Chile |  |  |  |  |
| 8 | Arbour et al. | 2015 | Canada |  |  |  |  |
| 9 | Arbour & Cook | 2006 | Canada |  |  |  |  |
| 10 | Bankoff & Perry | 2016 | Various or Global |  |  |  |  |
| 11 | Bardill | 2017 | Various or Global |  |  |  |  |
| 12 | Barker, C | 2020 | New Zealand |  |  |  |  |
| 13 | Barker, C | 2013 | New Zealand |  |  |  |  |
| 14 | Barker, J | 2004 | Various or Global |  |  |  |  |
| 15 | Beaton et al. | 2015 | New Zealand |  |  |  |  |
| 16 | Begay et al. | 2019 | United States |  |  |  |  |
| 17 | Benjamin | 2015 | Various or Global |  |  |  |  |
| 18 | Berg-Nordlie | 2022 | Norway |  |  |  |  |
| 19 | Birenbaum-Carmeli | 2004 | Various or Global |  |  |  |  |
| 20 | Blacksher et al. | 2022 | United States |  |  |  |  |
| 21 | Blacksher et al. | 2021 | United States |  |  |  |  |
| 22 | Bolleddula et al. | 2022 | Caribbean |  |  |  |  |
| 23 | Bonfim et al. | 2023 | Brazil |  |  |  |  |
| 24 | Boscarino et al. | 2022 | United States |  |  |  |  |
| 25 | Boyer et al. | 2011 | United States |  |  |  |  |
| 26 | Brief et al. | 2012 | United States |  |  |  |  |
| 27 | Burhansstipanov et al. | 2002 | United States |  |  |  |  |
| 28 | Burhansstipanov et al. | 2001 | United States |  |  |  |  |
| 29 | Burhansstipanov et al. | 2001 | United States |  |  |  |  |
| 30 | Burke et al. | 2022 | United States |  |  |  |  |
| 31 | Cabrera et al. | 2015 | Canada |  |  |  |  |
| 32 | Callaway | 2011 | Australia |  |  |  |  |
| 33 | Callaway | 2017 | South Africa |  |  |  |  |
| 34 | Capocasa & Volpi | 2019 | Italy |  |  |  |  |
| 35 | Caron et al. | 2023 | Canada |  |  |  |  |
| 36 | Caron et al. | 2020 | Various or Global |  |  |  |  |
| 37 | Carroll et al. | 2022 | United States |  |  |  |  |
| 38 | Carroll et al. | 2022 | United States |  |  |  |  |
| 39 | Chappell et al. | 2024 | Various or Global |  |  |  |  |
| 40 | Ching | 1997 | Various or Global |  |  |  |  |
| 41 | Chung-Li | 2010 | Taiwan |  |  |  |  |
| 42 | Claw et al. | 2021 | United States |  |  |  |  |
| 43 | Claw et al. | 2018 | Various or Global |  |  |  |  |
| 44 | Crampton & Parker | 2007 | New Zealand |  |  |  |  |
| 45 | Crigger | 1996 | Various or Global |  |  |  |  |
| 46 | Cunningham | 1998 | United States |  |  |  |  |
| 47 | D'Angelo et al. | 2020 | Various or Global |  |  |  |  |
| 48 | de Smith et al. | 2024 | Latin America |  |  |  |  |
| 49 | de Vries et al. | 2012 | South Africa |  |  |  |  |
| 50 | Dickenson | 2004 | Various or Global |  |  |  |  |
| 51 | Dodson & Williamson | 1999 | Australia |  |  |  |  |
| 52 | Dodson | 2000 | Australia |  |  |  |  |
| 53 | Dog | 1999 | Various or Global |  |  |  |  |
| 54 | Dubowsky et al. | 2023 | Australia |  |  |  |  |
| 55 | Dukepoo | 1999 | United States |  |  |  |  |
| 56 | Easteal, et al. | 2020 | Australia |  |  |  |  |
| 57 | Evans | 2012 | New Zealand |  |  |  |  |
| 58 | Fong et al. | 2004 | United States |  |  |  |  |
| 59 | Foster et al. | 1998 | United States |  |  |  |  |
| 60 | Foster et al. | 1998 | United States |  |  |  |  |
| 61 | Foster et al. | 1999 | United States |  |  |  |  |
| 62 | Fowler-Woods et al. | 2021 | United States |  |  |  |  |
| 63 | Fox | 2020 | Various or Global |  |  |  |  |
| 64 | Frost | 2022 | United States |  |  |  |  |
| 65 | Garrison et al. | 2020 | Various or Global |  |  |  |  |
| 66 | Garrison et al. | 2019 | United States |  |  |  |  |
| 67 | Garrison & Carroll | 2023 | United States |  |  |  |  |
| 68 | Garrison et al. | 2019 | United States |  |  |  |  |
| 69 | Garrison | 2013 | United States |  |  |  |  |
| 70 | Gillett & McKergow | 2007 | New Zealand |  |  |  |  |
| 71 | Gillett & Tamatea | 2012 | New Zealand |  |  |  |  |
| 72 | Gonzales et al. | 2018 | United States |  |  |  |  |
| 73 | Greely | 1999 | United States |  |  |  |  |
| 74 | Gulyaeva | 2022 | Various or Global |  |  |  |  |
| 75 | Haring et al. | 2021 | United States |  |  |  |  |
| 76 | Haring et al. | 2018 | Various or Global |  |  |  |  |
| 77 | Harry | 1996 | Various or Global |  |  |  |  |
| 78 | Hay | 2018 | Canada |  |  |  |  |
| 79 | Hayden | 2007 | United States |  |  |  |  |
| 80 | Hayes | 2011 | Various or Global |  |  |  |  |
| 81 | Hermes et al. | 2021 | Australia |  |  |  |  |
| 82 | Hiratsuka et al. | 2020 | United States |  |  |  |  |
| 83 | Hudson et al. | 2020 | Various or Global |  |  |  |  |
| 84 | Hudson et al. | 2016 | New Zealand |  |  |  |  |
| 85 | Hudson et al. | 2007 | New Zealand |  |  |  |  |
| 86 | Hull | 2019 | United States |  |  |  |  |
| 87 | Ilkilic & Norbert | 2009 | United States |  |  |  |  |
| 88 | Jacobs et al. | 2010 | Various or Global |  |  |  |  |
| 89 | James et al. | 2014 | United States |  |  |  |  |
| 90 | Johnson et al. | 2009 | United States |  |  |  |  |
| 91 | Kaladharan et al. | 2021 | Australia |  |  |  |  |
| 92 | Kamaara & Campbell | 2020 | Africa |  |  |  |  |
| 93 | Dalton | 2004 | United States |  |  |  |  |
| 94 | Kent | 2013 | Peru |  |  |  |  |
| 95 | Khan | 1999 | United States |  |  |  |  |
| 96 | Koloi-Keaikitse et al. | 2024 | South Africa |  |  |  |  |
| 97 | Kolopenuk | 2020 | Canada |  |  |  |  |
| 98 | Kowal et al. | 2017 | Australia |  |  |  |  |
| 99 | Kowal et al. | 2012 | Australia |  |  |  |  |
| 100 | Kowal et al. | 2015 | Australia |  |  |  |  |
| 101 | Kowal | 2015 | Australia |  |  |  |  |
| 102 | Kowal | 2013 | Australia |  |  |  |  |
| 103 | Kowal & Anderson | 2012 | Australia |  |  |  |  |
| 104 | Lara-Riegos & Azcorra-Pérez | 2023 | Mexico |  |  |  |  |
| 105 | Lavoie et al. | 2024 | Canada |  |  |  |  |
| 106 | Lee et al. | 2001 | Various or Global |  |  |  |  |
| 107 | Lee | 2021 | Various or Global |  |  |  |  |
| 108 | MacIntosh | 2005 | United States |  |  |  |  |
| 109 | Mathew et al. | 2017 | Various or Global |  |  |  |  |
| 110 | McInerney-Leo et al. | 2020 | Australia |  |  |  |  |
| 111 | McWhirter & Savulescu | 2015 | Australia |  |  |  |  |
| 112 | McWhirter et al. | 2012 | Australia |  |  |  |  |
| 113 | McWhirter et al. | 2013 | Australia |  |  |  |  |
| 114 | Merriman & Cameron | 2007 | New Zealand |  |  |  |  |
| 115 | Minaya & Roque | 2015 | Peru |  |  |  |  |
| 116 | Mizuno et al. | 2023 | Laos |  |  |  |  |
| 117 | Montoya | 2007 | United States |  |  |  |  |
| 118 | Morgan et al. | 2019 | Canada |  |  |  |  |
| 119 | Mudd-Martin et al. | 2021 | Various or Global |  |  |  |  |
| 120 | Munsterhjelm | 2015 | Brazil |  |  |  |  |
| 121 | Nasir et al. | 2022 | Australia |  |  |  |  |
| 122 | Nature publishing group | 2004 | United States |  |  |  |  |
| 123 | Nothling-Slabbert & Pepper | 2010 | South Africa |  |  |  |  |
| 124 | Nowrouzi et al. | 2016 | Canada |  |  |  |  |
| 125 | O'Connell | 2007 | Australia |  |  |  |  |
| 126 | Ogunrin et al. | 2022 | West Africa |  |  |  |  |
| 127 | Ortiz-Prado et al. | 2020 | South America |  |  |  |  |
| 128 | Paniagua & Taylor | 2008 | United States |  |  |  |  |
| 129 | Paradies et al. | 2007 | Various or Global |  |  |  |  |
| 130 | Paz Reverol et al. | 2016 | Venezuela |  |  |  |  |
| 131 | Peñas-Lledó et al. | 2020 | New Zealand |  |  |  |  |
| 132 | Pellekaan | 2011 | Australia |  |  |  |  |
| 133 | Perbal | 2013 | New Zealand |  |  |  |  |
| 134 | Prictor et al. | 2020 | Australia |  |  |  |  |
| 135 | Pullman & Nicholas | 2012 | Various or Global |  |  |  |  |
| 136 | Radin, J | 2014 | Various or Global |  |  |  |  |
| 137 | Radin, J.; Kowal, E. | 2015 | Various or Global |  |  |  |  |
| 138 | Reardon, Jenny | 2001 | United States |  |  |  |  |
| 139 | Reedy et al. | 2020 | United States |  |  |  |  |
| 140 | Reis et al. | 2023 | Australia |  |  |  |  |
| 141 | Ridgeway et al. | 2019 | United States |  |  |  |  |
| 142 | Rigden, Pam | 1997 | Various or Global |  |  |  |  |
| 143 | Robertson et al. | 2018 | New Zealand |  |  |  |  |
| 144 | Rodriguez et al. | 2022 | Philippines |  |  |  |  |
| 145 | Rogers-LaVanne et al. | 2023 | United States |  |  |  |  |
| 146 | Rotimi & Marshall | 2010 | Various or Global |  |  |  |  |
| 147 | Rupert et al. | 2003 | Mexico |  |  |  |  |
| 148 | Rusert & Royal | 2011 | United States |  |  |  |  |
| 149 | Sahota | 2014 | United States |  |  |  |  |
| 150 | Sahota | 2012 | United States |  |  |  |  |
| 151 | Samarasinghe et al. | 2023 | Australia |  |  |  |  |
| 152 | Santos | 2008 | Various or Global |  |  |  |  |
| 153 | Schofield et al. | 2011 | Australia |  |  |  |  |
| 154 | Schwartz-Marin & Restrepo | 2013 | Various or Global |  |  |  |  |
| 155 | Scott et al. | 2005 | New Zealand |  |  |  |  |
| 156 | Sharp & Foster | 2007 | United States |  |  |  |  |
| 157 | Sharp & Foster (a) | 2002 | United States |  |  |  |  |
| 158 | Sharp & Foster (b) | 2002 | United States |  |  |  |  |
| 159 | Silcocks et al. | 2023 | Australia |  |  |  |  |
| 160 | Silva et al. | 2022 | Chile |  |  |  |  |
| 161 | Skantharajah et al. | 2023 | Various or Global |  |  |  |  |
| 162 | Nothling-Slabbert | 2011 | South Africa |  |  |  |  |
| 163 | Soares et al | 2023 | Australia |  |  |  |  |
| 164 | Stevenson et al. | 2013 | Canada |  |  |  |  |
| 165 | Swazo, N.K. | 2005 | United States |  |  |  |  |
| 166 | Taitingfong et al. | 2020 | United States |  |  |  |  |
| 167 | Taiwo et al. | 2020 | Africa |  |  |  |  |
| 168 | Tallman et al. | 2024 | Peru |  |  |  |  |
| 169 | Taniguchi et al. | 2012 | Various or Global |  |  |  |  |
| 170 | Taupo | 2012 | New Zealand |  |  |  |  |
| 171 | Tone-Pah-Hote & Redvers | 2022 | United States |  |  |  |  |
| 172 | Tong et al. | 2020 | Australia |  |  |  |  |
| 173 | Trinidad et al. | 2015 | Various or Global |  |  |  |  |
| 174 | Tucker et al. | 2006 | United States |  |  |  |  |
| 175 | Tupara | 2012 | New Zealand |  |  |  |  |
| 176 | Underkuffler | 2007 | United States |  |  |  |  |
| 177 | Valiani et al. | 2023 | Various or Global |  |  |  |  |
| 178 | Valiani | 2022 | Canada |  |  |  |  |
| 179 | Van Holst Pellekaan | 2012 | Australia |  |  |  |  |
| 180 | Vawer et al. | 2013 | United States |  |  |  |  |
| 181 | Ventura Santos | 2002 | Brazil |  |  |  |  |
| 182 | Waanders et al. | 2023 | Various or Global |  |  |  |  |
| 183 | Weijer | 2000 | Canada |  |  |  |  |
| 184 | Weijer & Anderson | 2002 | Various or Global |  |  |  |  |
| 185 | Wensley & King | 2008 | New Zealand |  |  |  |  |
| 186 | Whittle | 2010 | New Zealand |  |  |  |  |

Appendix 2

Table 2: Summary of International, national, local and First Nation(s) laws, legislations and frameworks pertinent to genomics research involving Indigenous Peoples.

| **Legal Considerations** | |
| --- | --- |
| **International laws, legislations and frameworks pertinent to genomics research** | |
| The Universal declaration on the human genome and human rights is an international framework that ensure advances in genetics/genomics are used in ways that respect human dignity and rights (66). The declaration outlines principles of human dignity and rights as they relate to the human genome. It encourages international cooperation to support health and research, protects again misuse, prohibits financial profit from an unaltered human genome and advocates for establishment of appropriate legislative, administrative, and educational measures (33,66).  Complementing this is the broader, United Nations Declaration on the Rights of Indigenous Peoples (UNDRIP) which emphasises Indigenous Peoples ' rights to maintain, control, protect, and develop their cultural heritage, traditional knowledge, and genetic resources, including protection of genetic data (2). | |
| **National laws, legislations and frameworks pertinent to genomics research**  It is important to recognise that some Indigenous communities have developed their own legal or ethical frameworks governing research which operate alongside national and international laws to protect community interests and uphold sovereignty. | |
| **Australia** | The Aboriginal and Torres Strait Islander Social Justice Commissioner advocates for the protection of Indigenous genetic information in line with the 1997 Convention on Biological Diversity (CBD), a treaty advocating for informed consent and fair and equitable benefit-sharing, including financial and social (6,43). In Australia, research involving Aboriginal and Torres Strait Islander people has to adhere to the National Health and Medical Research Council (NHMRC) research guidelines which emphasise ethical conduct in research involving Aboriginal and Torres Strait Islander peoples (67). |
| **New Zealand** | The Treaty of Waitangi (Te Tiriti o Waitangi), signed in 1840 establishes the principles of partnership, protection, and participation between Māori and the British Crown and continues to underpin current legislation and research frameworks protecting the rights and interests of Māori people (15,23,68). In line with the treaty, genomics research conducted with and for Māori must ensure that their interests and rights are respected. Summarised by Tupara, 2012, “it is both a human right and a Treaty of Waitangi obligation for a research participant to be able to engage as an equal partner with researchers in decision making” (69). In addition to this treaty, the NZ Health Research Council provides guidelines for conducting health research involving Māori and promotes respect for Māori cultural values, obtaining informed consent, and ensuring equitable benefit-sharing (23). |
| **Canada** | The Tri-Council Policy Statement: Ethical Conduct for Research Involving Humans which addresses research involving Indigenous Peoples and emphasises the respect for their rights and community involvement in research processes (44). Additionally, the First Nations Health Authority ensures health research, including genomics research, is conducted in line with community needs and priorities (37) and the Canadian Institutes of Health Research Guidelines for Health Research Involving Aboriginal People are comprehensive and are model utilised by other countries and promotes the need for researches to develop relationships with communities and understand their worldviews (3,70). These guidelines detail “re-consent for multiple uses of samples, acknowledgment of intellectual property rights, protection of indigenous rights in cultural and sacred knowledge as well as recognition of ownership and stewardship of data or biological samples.”(70) |
| **United States** | There are over 500 recognised Native American tribes. Research involving Native American communities may be reviewed by the Indian health Service (IHS) and IHS Institutional Review Boards (IRBs) which are federal entities that ensure ethical and culturally appropriate conduct. Some tribes have established tribal-specific regulations to protect group members. For example, the Navajo Nation Human Research Code, a set of tribal regulations governs research conducted on Navajo land or involving Navajo people and requires review and approval by the Navajo Nation Human Research Review Board which emphasises informed consent, cultural sensitivity, and benefit-sharing (5,42). Researchers should be aware that compliance with both federal and tribal requirements may be necessary, depending on the context. |

66. Tong SYC, D’Antoine H, McKinnon M, Turner K, Hudson M, Brown N, et al. Lessons learned in genetic research with Indigenous Australian participants. Med J Aust. 2020;212(5):200-202.e1.

67. Prictor M, Huebner S, Teare HJA, Burchill L, Kaye J. Australian Aboriginal and Torres Strait Islander Collections of Genetic Heritage: The Legal, Ethical and Practical Considerations of a Dynamic Consent Approach to Decision Making. J Law Med Ethics. 2020;48(1):205–17.

68. Barker C. “The Ancestors Within”; Genetics, Biocolonialism, and Medical Ethics in Patricia Grace’s Baby No-Eyes. Journal of Literary & Cultural Disability Studies. 2013;7(2):141–58.

69. Tupara H. Ethics and Health Research: Decision Making in Aotearoa New Zealand. AJOB Prim Res. 2012;3(4):40–52.

70. Jacobs B, Roffenbender J, Collmann J, Cherry K, Bitsói LL, Bassett K, et al. Bridging the Divide between Genomic Science and Indigenous Peoples. J Law Med Ethics. 2010 Sept;38(3):684–96.
